# 

**Published:** 1912-07

**Authors:** 


### CONTENTS.

ORIGINAL COMMUNICATIONS:—

Address of Welcome. By Hon. Thomas H. Jeffries, Atlanta, Ga. ---- 171
The Chattahoochee Valley Medical and Surgical Association. By J. S. Todd, Atlanta, Ga. ---- 174
Some Observations on the Use of Apomorphine. By Dr. J. S. Horsley, West Point, Ga. ---- 180
The Eye as an Index to Disease. By Park Howell, Atlanta, Ga. ---- 186
Pin-Point Os Uteri. By Dr. Jno. Prather, Ft. Davis, Ala. ---- 189
The Chronic Pains of the Chest. By Dr. Jno. Prather, Ft. Davis, Ala. ---- 195
Personal Observations upon the Effects and Treatment of Chronic Constipation. By Lewis M. Gaines, A. B., M. D., Atlanta, Ga. ---- 202

EDITORIAL ---- 207

NEWS AND NOTES ---- 207

